# Real‐Time Tool Detection in Laparoscopic Datasets for Surgical Training in Low‐Resource Settings

**DOI:** 10.1049/htl2.70045

**Published:** 2025-12-10

**Authors:** Omar Choudhry, Sharib Ali, Chandra Shekhar Biyani, Dominic Jones

**Affiliations:** ^1^ School of Computer Science University of Leeds Leeds UK; ^2^ St Jamess University Hospital Leeds Teaching Hospitals NHS Trust Leeds UK; ^3^ School of Electronic and Electrical Engineering University of Leeds Leeds UK

**Keywords:** computer vision, data acquisition, image processing, learning (artificial intelligence), surgery

## Abstract

In low‐resource settings, there is a critical need for skilled surgeons. Alternative training processes that include computer‐assisted surgical skill evaluation are essential to address this gap. Using tool detection, surgical videos can be leveraged to derive insights into surgical skill assessment. However, state‐of‐the‐art laparoscopic tool detection methods usually have more complex architectures tailored for in vivo data, which suffer from challenges such as smoke, occlusion, bleeding, etc., which are absent from in vitro training contexts. Thus, this paper tests multiple anchor‐based and anchor‐free, convolution‐ and transformer‐based, traditional (non‐surgical domain‐specific) computer vision deep learning state‐of‐the‐art models. With various hardware configurations on a newly curated in‐house laparoscopic box‐trainer dataset, we emphasise real‐time performance on low‐cost embedded devices. Overall, the anchor‐free YOLOv8‐X model was the most accurate, achieving ${\mathrm{mAP}}_{50}$ of 99.5% and ${\mathrm{mAP}}_{50:95}$ of 96.6% with an inference time of 23.5 ms/≈42.6 FPS on an NVIDIA Jetson Orin Nano 8GB (comparable low‐cost hardware which could be expected to run real‐time skill assessment methods for surgical training boot camps in a resource‐constrained environment). The most efficient model was YOLOv11‐N, providing 3.1 ms/≈322.6 FPS with a performance difference of +0% ${\mathrm{mAP}}_{50}$ and –2.1% ${\mathrm{mAP}}_{50:95}$. The results highlight the models' potential for effective real‐time detection of surgical tools and are suitable for further downstream assessment of surgical skills, even in resource‐constrained environments.

## Graphical abstract

This paper explores the potential for computer‐assisted evaluation of surgical skill in low‐resource training settings by detecting and tracking tools in laparoscopic box‐trainer videos. It introduces a new in‐house dataset and systematically tests diverse state‐of‐the‐art detection approaches across multiple hardware configurations with emphasis on real‐time performance on affordable embedded devices. The findings show that these methods can deliver accurate and efficient tool detection suitable for downstream skill assessment in resource‐constrained environments.

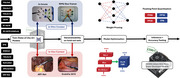

## 1 Introduction

Minimally invasive surgery (MIS), particularly laparoscopy, significantly reduces tissue damage, blood loss, infection rates, and recovery times compared to open surgery, resulting in better patient outcomes and reduced cost, making it highly beneficial for resource‐constrained environments (RCEs) [1]. However, adoption in RCEs is severely limited due to the scarcity of skilled surgeons, high equipment costs, and challenges related to the quality and availability of surgical training [2]. The Lancet Commission on Global Surgery highlights a critical global shortage of surgical services, affecting approximately 5 billion people and requiring an additional 143 million surgeries annually [3]. A significant portion of the global shortfall of 44.5 million healthcare workers identified by the WHO is in the surgical sector, exacerbating disparities and poor surgical outcomes in low‐ and middle‐income countries (LMICs) [4]. To overcome these challenges, automated AI‐driven surgical skill assessment systems offer objective, consistent, and scalable solutions to increase the throughput of surgical trainees. Traditional manual assessment methods, performed by expert surgeons, are subjective, time‐consuming, and prone to human bias [5]. AI‐based computer vision technologies capable of real‐time surgical tool detection, tracking and performance evaluation hold promise for enhancing surgical training, addressing skill gaps and improving patient outcomes in LMICs. This study specifically focuses on the application of state‐of‐the‐art (SOTA) object detection models in a new domain, namely, surgical tool detection on an in‐house dataset of surgical training videos. This includes an investigation into generalizability and inference speed in the context of low‐resource settings—with the aim of providing insights into surgical tool detection methods for automated surgical training skill assessment techniques in low‐resource settings. Though we do not propose any methodological innovations, this work's novelty lies in systematic benchmarking and deployment of SOTA detectors to determine, under controlled and reproducible real‐world conditions, how existing architectures of very different sizes behave when compiled and deployed on low‐cost hardware. This work provides the first systematic benchmark of SOTA anchor‐based and anchor‐free object detection models for laparoscopic tool detection, explicitly targeting deployment in RCEs. We compare performance across four datasets (in‐house, WMU, ART‐Net and EndoVis), analyse cross‐domain generalisation gaps, and quantify real‐time feasibility on an NVIDIA Jetson Orin Nano edge‐computing device. These results establish practical deployment baselines and inform future integration of tool detection into automated surgical skill assessment.

## 2 Related Work

### 2.1 State‐of‐the‐Art Detection Models in Computer Vision

#### 2.1.1 Convolutional Architectures

SOTA object detection has advanced rapidly since the inception of modern deep learning, beginning with convolutional neural network architectures. Although currently, a few high‐performing model families dominate this space. Whilst the R‐CNN (introduced in 2013) two‐stage detectors series and its derivatives, for example, faster R‐CNN (2015), had long set the standard for accuracy, one‐stage models have closed the gap with more efficient designs—no longer relying on region proposals and hand‐tuned anchors. [6, 7]. Nevertheless, whilst two‐stage detector frameworks can excel in general settings, they tend to be less adaptable to complex scenes and depend on large labelled datasets [8]. Modern one‐stage detectors, such as the YOLO (You‐Only‐Look‐Once) series, continue to push SOTA performance on traditional computer vision datasets. YOLOv5 (2020) established a strong baseline with an efficient CSP‐Darknet backbone [9]. YOLOv7 (2022) surpassed all prior comparative detectors, reaching SOTA performance while maintaining real‐time speeds, using extended efficient layer aggregation networks [10]. YOLOv8 (2023) introduced a fully anchor‐free detection pipeline, moving away from the manual anchor boxes used in earlier YOLO versions [11]. This transition to an anchor‐free design simplified training by reducing the number of hyperparameters and improved adaptability to objects of varying sizes. The latest convolution‐based versions, YOLOv10 (early 2024) and YOLOv11 (late 2024), incorporated NMS‐free training (eliminating the costly non‐max suppression step), attention mechanisms and large‐kernel convolutional heads, yielding SOTA accuracy with lower latency [12, 13]. Amongst other SOTA one‐stage detectors include RetinaNet (2017), a breakthrough that introduced focal loss to address class imbalance, achieving accuracy rivalling that of two‐stage methods while maintaining fast inference [14]. EfficientDet (2020) further improved the speed‐accuracy trade‐off by employing a compound scaling strategy and optimised feature fusion (BiFPN), producing a family of models that range from mobile‐friendly to SOTA accuracy [15]. Convolutional architectures have been shown to be more efficient than transformer‐based models due to their less memory‐intensive attention mechanisms. Even though innovations that allow faster inference, such as FlashAttention, exist, they are not widely available on all edge‐computing devices due to hardware requirements.

#### 2.1.2 Transformer‐Based Models

Whilst convolution‐based models have long represented the SOTA, their capacity to capture relationships between distant features within an image is inherently limited by the locality of convolutional kernels. In contrast, transformer‐based architectures employ global attention mechanisms, enabling them to model long‐range dependencies and learn feature correlations across the entire image, thereby enhancing overall image understanding. More recently, transformer‐based detectors have risen to prominence, spearheaded by the DETR (DEtection TRansformer) (2020) framework, which reconceptualises detection as a direct set prediction task without the need for hand‐crafted anchors or non‐maximum suppression [16]. This results in an end‐to‐end approach, removing hand‐crafted post‐processing by using bipartite matching to output final boxes directly. In general, transformer‐based architectures have much higher latency, though some models claim real‐time performance. Building on DETR, the RT‐DETR (2024) family (v1–v3) advances end‐to‐end real‐time detection by (v1) introducing an efficient hybrid encoder for fast multi‐scale processing with uncertainty‐minimal query selection, (v2) adding a ‘bag‐of‐freebies’ training recipe with selective multi‐scale deformable attention and a deployment‐friendly discrete sampling operator, and (v3) enriching supervision via training‐only hierarchical dense positive supervision while preserving latency [17]. Concurrently, RF‐DETR (2025) leverages robust features learnt from a fully self‐supervised DINOv2 (2023) backbone [18] and integrates deformable/LW‐DETR (light‐weight) design choices to enhance global context modelling and domain robustness in an end‐to‐end, NMS‐free detector [19]. YOLOv12 (2025) broke away from the traditional convolution‐based architectures of the YOLO family, introducing an attention‐centric redesign that integrates efficient transformer‐style attention modules into the backbone and detection head, thereby exceeding the accuracy of prior models at a slightly slower but comparable speed [20]. The most recent DINOv3 (2025) self‐supervised backbones provide stronger dense features. The authors provide benchmarks using a DETR detection adapter trained on top of a DINOv3 backbone to obtain competitive accuracy with minimal task‐specific tuning [21]. Compared to convolutional detectors, these transformer‐based variants achieve superior global context modelling and accuracy in cluttered or occluded scenes, yet they typically incur higher computational cost and longer convergence times, making their deployment less feasible in embedded or RCEs.

### 2.2 Laparoscopic Instrument Detection

Real‐time detection of surgical tools in laparoscopic videos is a fundamental step for understanding procedures and enabling advanced analyses [22]. Surgical tool detection research has found that anchor‐based and proposal‐based methods fail to detect surgical tools in real‐time. Thus, recent works favour one‐stage architectures for surgical data, leveraging their speed and flexibility. Attention mechanisms have been incorporated into laparoscopic instrument detection models, achieving improved accuracy [23]. Efforts have been made to improve the real‐time performance of the original DETR, which suffered from slow training convergence, requiring large datasets, and extensive training data, as well as substantial computational resources, creating the dense‐transformer network, DTX (2024) [22]. Nevertheless, some works still use the robust Faster‐CNN model with a ResNet backbone [24], or even the older YOLOv5 [25]. This contrast highlights a trade‐off in the surgical domain: older CNN‐based models (e.g., Faster R‐CNN, YOLOv5) remain appealing for their stability and ease of use, whereas newer transformer‐inspired approaches (e.g., DTX, RT‐DETR) promise higher accuracy but require significantly greater computational resources and training data. Beyond per‐frame detection, tracking algorithms link instrument detections over time to produce trajectories. Many recent trackers utilise a detection backbone with YOLO to provide bounding boxes in each frame, and then apply data association for identity tracking. For instance, one approach combines YOLO‐based detection with the ByteTrack multi‐object tracking method to achieve robust real‐time instrument tracking [26]. Similarly, the SurgiTrack [27] system utilises YOLOv7 for precise tool detection and an attention mechanism to model each instrument's motion direction, which aids in re‐identifying occluded tools and achieves state‐of‐the‐art multi‐tool real‐time tracking performance on the CholecTrack20 dataset. Taken together, YOLO‐based backbones dominate downstream tasks because they combine real‐time performance with sufficient accuracy, while DETR‐style or attention‐heavy architectures have yet to demonstrate the same balance for continuous laparoscopic video streams. Recent work on automated skill assessment has utilised YOLOv5, trained on the Cholec50 dataset, as the baseline tool detection method [28]. In some work, rather than using off‐the‐shelf detection models, backbones such as ResNet [29, 30] or VGG [31] are used instead. [32]

### 2.3 Clinical Translation in Low‐Resource Settings

RCEs may suffer from real purchase costs, power requirements, internet speed and availability [33]. Many of the SOTA detection methods, especially transformer‐based architectures, only exhibit real‐time performance and high accuracy on computationally expensive hardware—generally unavailable in low‐resource settings [34]. YOLOv7‐RepFPN (2024) was specifically designed for use in embedded systems within the operating room. By halving feature channels and using efficient re‐parameterisation blocks, it retains high accuracy while drastically reducing computation [35]. It achieved ≈63 FPS on a modest device, nearly 50% faster than vanilla YOLOv7 (≈42 FPS) with only a ≈1% drop in accuracy. This trade‐off is crucial to meet real‐time requirements under limited compute. Similarly, choosing smaller model variants can balance accuracy and latency; using the medium‐variant of the YOLOv8 model was found to offer a good compromise for real‐time instrument detection at ≈45–60 FPS with high precision, when evaluating surgeons on a cadaver in a real‐world environment [36]. Thus, while transformer‐based methods show promise in high‐resource settings, CNN‐based YOLO variants remain the most viable option for RCEs, where embedded deployment and energy efficiency are prioritised over accuracy gain trade‐offs.

## 3 Datasets

### 3.1 Overview of Surgical Instrument Datasets

**FIGURE 1:**
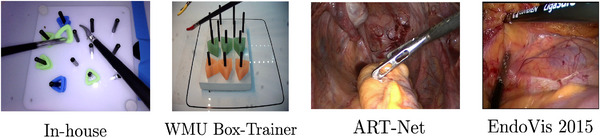
Sample dataset images. For the in vitro training context we use the in‐house and WMU Box‐Trainer datasets (both of laparoscopic peg transfer). For the in vivo surgical context we use the ART‐Net dataset for laparoscopic hysterectomy and the EndoVis 2015 dataset for laparoscopic colorectal. Images are cropped to fit and not to scale).

High‐quality datasets are essential for developing and evaluating accurate and generalisable tool detection methods to be used in laparoscopic videos from low‐resource settings. In MIS, various public datasets have been released to facilitate computer vision research on instrument detection, tracking, segmentation, phase recognition, and tool pose estimation [37]. These datasets typically consist of endoscopic video frames annotated with instrument locations (e.g., bounding boxes or pixel masks) and occasionally other information, such as tool type or surgical phase. They have enabled significant advances in surgical data science, including improved instrument detection accuracy with deep learning models. However, most existing datasets focus on data from high‐resource settings (e.g., well‐equipped surgical centres) and often involve robotic surgical systems or high‐resolution laparoscopic imaging. Collecting similar datasets in low‐resource settings presents additional challenges. RCEs may lack expensive laparoscopic trainers or recording infrastructure, making data acquisition difficult. Furthermore, manual annotation of videos requires expert time, which may be scarce in low‐resource hospitals. As a result, there is a paucity of surgical tool datasets reflecting the conditions and constraints of low‐resource settings. Moreover, most datasets focus on in vivo procedures (within the human body) or cadavers and not in vitro surgical training tasks. However, curating such datasets is crucial for creating robust tool detection models that can be generalised to diverse training environments. This work considers four different datasets (including one collected in‐house) that span both in vivo and surgical training tasks (see Figure 1 and Table 1).

**TABLE 1:** Comparison of surgical instrument datasets. The in vitro datasets have a much larger number of labelled frames compared to the in vivo datasets. Resolution varies across all datasets. The ART‐Net and EndoVis 2015 datasets originally contained segmentation masks which were converted to regular bounding boxes as in the training datasets.

Dataset	Labelled frames	Resolution	Context	Tool annotation
In‐house dataset	3725	1280×720	Peg transfer	Bounding box
WMU Box‐Trainer [39]	3061	1600×1216, 1024×768	Peg transfer	Bounding box
ART‐Net [31]	635	1920×1080	Hysterectomy	Segmentation masks
EndoVis 2015 [40]	340	640×480	Colorectal	Segmentation masks

### 3.2 In‐House Dataset

The in‐house dataset was collected at the 8th Urology Simulation Boot Camp in Leeds, UK^1^. Recorded using a laparoscopic box trainer, it comprises 24 video trials each from a distinct urologist (14 novices/trainees and 10 experts/specialist clinicians), totalling 103,629 frames across ≈2 h of footage. Roughly 3700 frames were manually annotated with bounding boxes (though the full dataset included ground truth seven degrees‐of‐freedom motion data for location in 3D space and tooltip orientation, using a kinematic data collection setup). The dataset captures a peg transfer task^2^ using two laparoscopic tools (Short Fenestrated Grasper, Curved; Surgical Innovations, UK), under varied lighting conditions, in slightly lower quality (1280×720 pixels), reflecting the challenges typical in low‐resource settings. Out of the labelled frames, one video was entirely annotated (2680 frames) and used for validation (to ensure no leakage between the training and test sets). The test frames originate from a single continuous video with larger variability, incorporating camera position and focus changes, occlusion and luminance variability, with 2680 frames compared to the ≈1000 training set. There is more heterogeneity in the set this way, which is missable due to frame selection bias during annotation. Other training frames were selected by taking every 100th frame from all other videos. There is no class imbalance since almost all frames contain both tools and tooltips, or none at all.

### 3.3 WMU Box Trainer Dataset (Laparoscopic Training Videos)

The WMU Box Trainer dataset [39] is a collection of videos captured during laparoscopic training exercises on Fundamentals of Laparoscopic Surgery (FLS)‐style box trainer. The dataset consists of circle‐cutting, tooltip tracking and peg transfer videos. Images from the peg transfer videos were selected based on their context relevance. In total, there are 2842 training and 219 test labelled images, with various annotations including bounding boxes, tool classification, truncation, difficulty, in/out‐of‐field and position (on/off peg). Recorded from multiple camera perspectives (top, side and front), the images have resolutions of both 1600 ×1216 and 1024 ×768 pixels. All images were used for generalisability validation using models trained on the in‐house dataset.

### 3.4 ART‐Net Dataset (Laparoscopic Hysterectomy)

The ART‐Net dataset [31] originates from 29 clinical hysterectomy videos. Whilst 3000 images were annotated solely with tool presence and absence, it contains 508 and 127 annotated images for training and testing, respectively. These annotations include segmentation masks and geometric primitives (keypoints for the tool's tip, mid‐line and edges). Recorded in high definition (1920 ×1080 pixels) in an in vivo clinical setting, the dataset focuses on generic tool detection rather than fine‐grained classification. Its rich annotations have enabled multi‐task learning for segmentation and keypoint detection, though in this study, the segmentation masks are converted to bounding boxes for consistent evaluation across datasets.

### 3.5 EndoVis 2015 Dataset (Laparoscopic Colorectal)

The EndoVis 2015 dataset was part of the MICCAI Endoscopic Vision Instrument Segmentation and Tracking Sub‐Challenge [40]. It consists of images and videos for segmentation and tracking, with both rigid (in vivo laparoscopic colorectal surgeries) and robotic instruments (ex vivo sequences using a da Vinci instrument). However, the robotic data was not used due to this study's laparoscopic focus. Instead, both the segmentation (≈160) and tracking (≈180) images with available annotations were used. Originally, each frame was annotated with pixel‐level segmentation masks for surgical instruments, which distinguishes parts such as the shaft and tip, which were converted to bounding boxes. All ≈340 images were used for validation.

## 4 Methodology

### 4.1 Experiment Details

**FIGURE 2:**
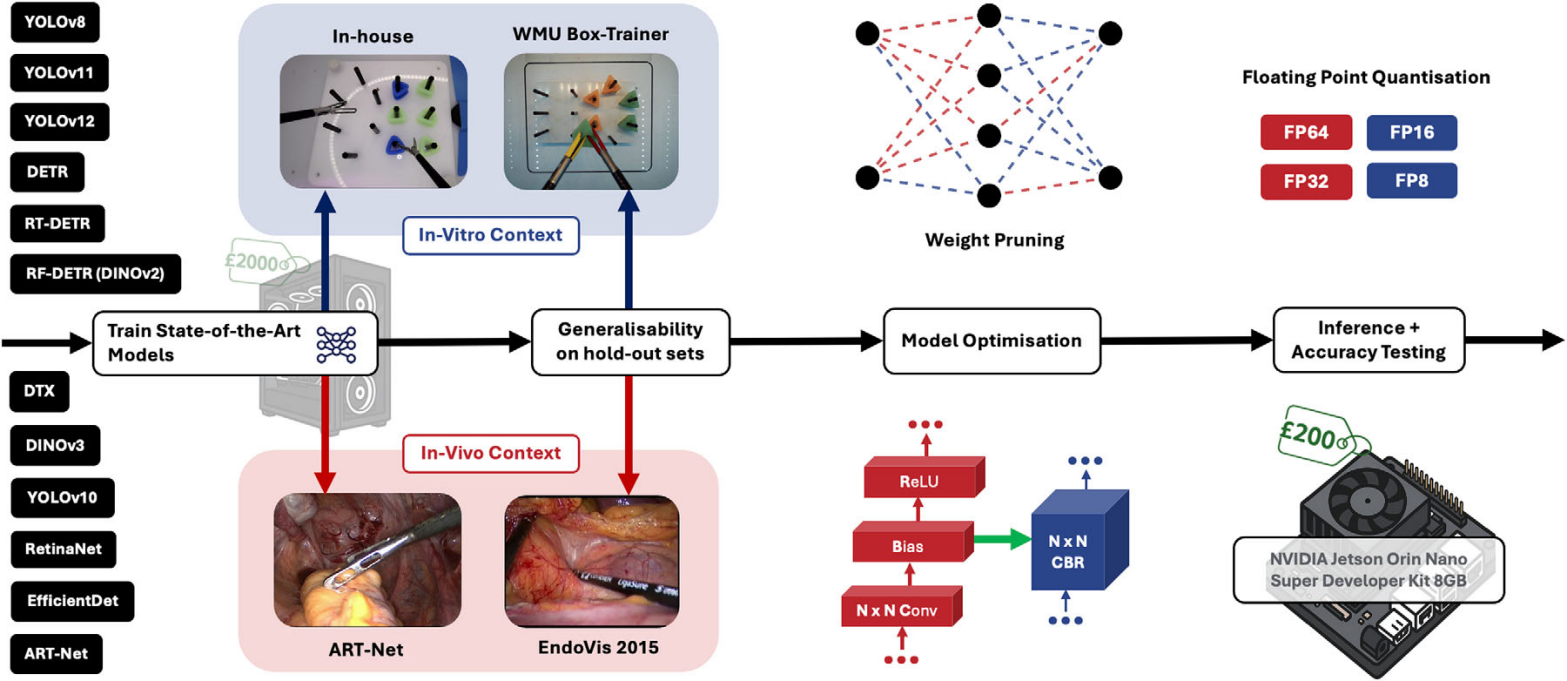
Pipeline for running experiments. We train all SOTA detectors on the in‐house and ART‐Net datasets for in vitro and in vivo contexts. We then perform a generalisability test on the hold‐out sets without additional training on the WMU Box‐Trainer and EndoVis 2015 datasets. We then perform model optimisation using TensorRT, including model compilation, weight pruning and floating‐point quantisation. This is compiled on the NVIDIA Jetson Orin Nano Super Developer Kit 8GB (edge‐computing device) for inference and accuracy testing as validation for real‐world deployment.

Multiple SOTA models were implemented and evaluated to address the resource constraints typical of low‐resource settings, followed by generalisability and model optimisation experiments (Figure 2). Initial model training and validation were conducted on a system configured with an AMD Ryzen 3900X CPU, 32 GB RAM, and an NVIDIA GeForce RTX 3060 GPU (12 GB VRAM), with Python 3.9.5 and PyTorch 2.2.1+cu121. The aim was to demonstrate that these models can be successfully deployed on less powerful, cost‐effective hardware. Transfer learning was employed by fine‐tuning models from existing SOTA checkpoints to reduce training times and leverage multiclass, multi‐task learning. Models were fine‐tuned on the in‐house and ART‐Net datasets (for an in vitro and in vivo context) and further validated on the WMU and EndoVis 2015 datasets, respectively. Whilst the tools in the WMU dataset have distinct coloured tips, task and background (Figure 1), which could hinder detection performance without additional fine‐tuning, we recognise that variation in the data is a typical real‐world problem. Thus, we treated the WMU and the EndoVis 2015 datasets as out‐of‐domain distribution external validation sets for generalisability. In practice, the goal is to ensure these models can eventually be deployed on even less powerful and more affordable hardware. An NVIDIA Jetson Orin Nano Super Developer Kit 8GB (comparative hardware ≈$249, which would be representative at a surgical training boot camp in a low‐resource setting) was used to test the inference speed of these trained models.

### 4.2 Choice of Models

#### 4.2.1 Anchor‐Free Methods

Anchor‐free detection was evaluated using five different architectures from YOLOv8 and YOLOv12—nano (N), small (S), medium (M), large (L), and extra large (X) (see Table 2). This allows investigation into the trade‐offs between model size, inference speed, and detection accuracy. One of the main architectural shifts in YOLOv8 (relative to its YOLOv5 predecessor) is that it removes the predefined anchor boxes altogether, designed to be anchor‐free by default [11]. Instead, it relies on direct x,y,w,h (horizontal bounding box centre in x‐axis, vertical bounding box centre in y‐axis, bounding box width, and bounding box height) regression from each point on the feature map. This anchor‐free approach increases model complexity by using more parameters but often improves generalisation, especially for objects of varying aspect ratios. In addition, we implemented the ART‐Net model (renamed SIMO for single‐input‐multiple‐output^3^ to avoid confusion with the dataset of the same name), using both a ResNet50 and the original VGG16 backbone. SIMO was reimplemented from the author's original repository from TensorFlow into PyTorch, with modifications to the final layer to output bounding box annotations for left and right tools. We further trained the following transformer‐based anchor‐free models: DETR; RF‐DETR across the smallest three architectures (N, S and M); RT‐DETRv3 with various backbones including ResNet‐18 (20M parameters), ResNet‐34 (31M), and ResNet‐50 (42M); DTX (utilising a ResNet‐50 backbone with six encoder/decoder layers, 256 hidden dimensions, 100 queries); and DINOv3, where we freeze the smallest provided self‐supervised backbone (21M for ViT‐S/16) and fine‐tune the detection adaptor (100M). Whilst we include these models for comparison as more relevant SOTA models, they are expected to be slower than convolutional architectures.

**TABLE 2:** Parameter sizes across different model architectures. Many model families use a general nomenclature such as ‘nano’, ‘small’, ‘medium’, ‘base’, ‘large’ and ‘extra large’ to define different architecture configurations of the same model. For RT‐DETR, we label each of the different ResNet backbones with this nomenclature ourselves. Smallest and largest models have been highlighted in bold.

Architecture	YOLOv8	YOLOv10	YOLOv11	YOLOv12	RF‐DETR	RT‐DETR
Nano (n)	3.2M	**2.3M**	2.6M	2.5M	30.5M	20.0M
Small (s)	11.2M	7.2M	9.4M	9.1M	32.1M	31.0M
Medium (m)	25.9M	15.4M	20.1M	19.6M	33.7M	42.0M
Base (b)	—	19.1M	—	—	—	—
Large (l)	43.7M	24.4M	25.3M	26.5M	—	—
Extra Large (x)	**68.2M**	29.5M	56.9M	59.3M	—	—

#### 4.2.2 Anchor‐Based Methods

For anchor‐based detection, we again examined different architectures of YOLOv10 and YOLOv11 (Table 2 illustrates the increasing YOLO model complexity from N to X variants). While YOLOv8 and YOLOv12 are anchor‐free methods, YOLOv10 and YOLOv11 still follow an anchor‐based paradigm. YOLOv10 introduces an NMS‐free head (one‐to‐one matching) for end‐to‐end inference; it continues using grid ‘anchor points’ and anchor‐style assignment in its training objectives, that is, each location (anchor point) on the feature map corresponds to a bounding‐box prediction, preserving the anchor‐based design from earlier YOLO variants. As for YOLOv11, it introduces new components—C3k2 (a more efficient bottleneck with fewer parameters, having the ability to switch between deeper feature extraction) and C2PSA (convolutional block with parallel spatial attention). Its outputs are expanded to cover instance segmentation, oriented bounding boxes, pose estimation, object tracking and image classification whilst retaining the multianchor layout in the head. Consequently, the final detection head uses predefined anchor points during training and inference. We prioritised testing YOLO models due to their lightweight nano and small variants suited for embedded deployment. PP‐YOLOE‐S (late 2022) was excluded as YOLOv8 Small surpasses it in both accuracy (+1.8 AP) and latency (1.2 ms vs ≈3 ms), while YOLOv10/11 inherit PP‐YOLOE's optimisations [41]. For other anchor‐based method comparisons, RetinaNet [14] and EfficientDet [15] were also trained with their default configurations.

### 4.3 Model Training

All models were trained for a maximum of 300 epochs using a standardised protocol designed to maximise robustness and generalisability. Due to varying model memory requirements, batch sizes varied across models, dropping as low as 2 for some of the larger models, like DINOv3, but 16 for some of the smaller YOLO Nano‐variants. The optimiser choice was architecture‐dependent: AdamW with weight decay $1\times 10^{-4}$ for transformer‐based models (RT‐DETR, RF‐DETR, DINOv3), with separate learning rates for encoder ($1\times 10^{-5}$) and decoder ($1\times 10^{-4}$) components in RF‐DETR. YOLOv12 models utilised AdamW with momentum 0.937, weight decay $1\times 10^{-3}$, and warmup momentum 0.8 over 2 epochs. Input images were uniformly scaled to a resolution of 640×640 pixels, except for DINOv3 which required 224×224 due to ViT backbone constraints. Early stopping was enforced with a patience of 10 epochs, monitoring validation loss (threshold at $1\times 10^{-4}$), to prevent over‐fitting. During training, an Intersection over Union (IoU) threshold of 0.7 was used, and a dropout rate of 0.2 was applied as a regularisation measure. In addition, data augmentation techniques – including multi‐scale training (480–800 pixel range with 32‐pixel steps), rotations (±10°), shears (±0.2), perspective transforms (±0.0005), mixup (α=0.2 weighted combination of random image pairs), mosaics (4‐image composition with 1.0 probability), copy–paste (10% probability for synthetic instance augmentation) and flips (10% for vertical and 50% for horizontal)—were incorporated to enhance the models generalisability. Hardware‐specific optimisations included mixed‐precision training (AMP) for transformer models, gradient clipping (max_norm = 0.1) for training stability, and exponential moving average (EMA) with decay 0.9997 for RF‐DETR models, especially important given the similar characteristics of the training images. Training infrastructure utilised CUDA‐optimised implementations, disk caching for dataset management, and four parallel workers for data loading. Model checkpoints were saved every epoch with best model selection based on validation ${\mathrm{mAP}}_{50}$, enabling robust model selection and preventing catastrophic forgetting during extended training periods.

### 4.4 Model Compilation

After initial training and validation, YOLO models were deployed on the Jetson to assess inference performance under edge computing conditions. We compiled the trained models using TensorRT to optimise the models for real‐time inference on this embedded platform. TensorRT, an SDK by NVIDIA, allows for model optimisation, achieving high‐performance deep learning inference on NVIDIA GPUs, including those found in edge computing devices like the Jetson. It optimises trained neural networks for deployment, yielding significantly higher throughput and lower latency, essential for real‐time applications. TensorRT achieves this through various techniques, including graph optimisations like layer fusion (combining multiple operations into a single GPU kernel, reducing the overhead of launching individual operations, for example, a convolution layer followed by a bias addition and a ReLU activation can often be fused into a single operation), pruning weights whose magnitude is close to zero, and kernel selection (choosing the most efficient kernel for each layer based on the target GPU architecture and input data types). Furthermore, TensorRT supports floating‐point quantisation, allowing the reduction of precision in the network's weights and activations. The significance of FP32 (32‐bit floating point precision) and FP16 (16‐bit floating point precision) is that FP32 offers high precision and is typically used during model training, but demands more memory and computational resources. In contrast, FP16 provides lower precision, reducing memory footprint, and faster computation on compatible NVIDIA GPUs, often resulting in increased inference speed and throughput. While FP16 offers efficiency benefits over FP32, there's a potential trade‐off with accuracy. Thus, we experiment with both modes of quantisation^4^.

However, a limitation here is that due to the architectures of some of the models, we could not appropriately compile them using the TensorRT SDK, especially due to memory constraints on the NVIDIA Jetson, resulting in compiling some of the models being impossible. Due to the calibration performed during compilation, it was not possible to compile on another device and then evaluate on the Jetson itself. Thus, we only focused on the YOLO models, where there is adequate support for compilation through the Ultralytics Python library.

### 4.5 Evaluation Metrics

#### 4.5.1 Accuracy

Models must be quantitatively evaluated for performance comparison and to validate the generalisability of the methods. The standard COCO metric yields mean average precision (mAP) scores for detected tool bounding boxes. For more detail, intersection over union (IoU) is a metric used across various loss functions in the models and defined as the ratio of the intersection area to the union area of the two bounding boxes. An IoU score of 1 indicates perfect alignment, while 0 indicates no overlap. An IoU loss is defined as 1−IoU. mAP measures the average precision across all classes for all n images at different IoU thresholds. Specifically, ${\mathrm{mAP}}_{50}$ refers to the mAP at an IoU threshold of 0.5, meaning that a predicted bounding box is considered correct if its IoU with the ground truth is greater than 50% (see Equation 1). ${\mathrm{mAP}}_{50:95}$ is a more stringent metric that averages the precision (AP) across multiple IoU thresholds t from 0.5 to 0.95 in increments of 0.05 (see Equation 2)—the most widely used computer vision metric symbolic to a confidence interval.

#### 4.5.2 Inference

Models must also be quantitatively evaluated for inference performance. This can be defined as the time taken—usually in milliseconds (ms)—from after pre‐processing the image until the model produces an output (before any post‐processing). We do not consider end‐to‐end performance, as our context defines surgical tool detection as an initial step prior to downstream tasks. Due to the constraints of a low‐resource setting, the models must perform with some level of real‐time performance, which we benchmark at 30FPS (≈33.3 ms per frame) and 60 FPS (≈16.67 ms per frame). We can also use this to define ‘small models’ as those which surpass this threshold as an objective metric, rather than the less interpretable floating‐point operations per second.

## 5 Results and Discussion

### 5.1 Quantitative Analysis of Model Performance

The anchor‐free YOLOv8‐X model was the most accurate, achieving a ${\mathrm{mAP}}_{50}$ of 99.5% and a ${\mathrm{mAP}}_{50:95}$ of 96.6% on the test set with an inference time of 21.7 ms, approximately 46 frames‐per‐second (FPS), demonstrating its effectiveness in real‐time surgical tool detection. The most efficient model was YOLOv11‐N, achieving a ${\mathrm{mAP}}_{50}$ of 99.5% (±0%) and a ${\mathrm{mAP}}_{50:95}$ of 94.5% (−2.1%) at 1.7 ms/588 FPS (with only 2.6 million parameters and 5.2MB weights, ≈26× less than YOLOv8‐X at 131 MB).

Direct model comparisons show that there was not necessarily a correlation between higher accuracy and using a more complex architecture. It is possible that smaller networks generalised better and faster than larger models. Though the different YOLO versions vary in many other properties, which could be investigated further, we notice that they all perform very similarly. In fact, some of the results are so similar (especially those above 99%, within a single percentage point of each other) that some discrepancy in the results could be due to labelling bias. Even though it could be expected that the anchor‐free property of YOLOv8 could be better suited for detecting laparoscopic tools (whose aspect ratios can change drastically with varying orientations, positions and camera angles), we see no significant differences in performance. RetinaNet may have been an exception to this due to anchor‐box optimisation, which resulted in an almost perfect ${\mathrm{mAP}}_{50}$ score. The reimplemented SIMO vastly underperformed compared to the original results, its twin‐head design optimised for segmentation under‐utilises bounding‐box supervision, requiring keypoints (missing in our data, resulting in poorer performance). Likewise, many of the transformer‐based models did not outperform the convolution‐based methods. The DETR's low recall could be attributed to its slow bipartite‐matching convergence on small objects and requiring large‐scale data to converge, which our dataset cannot provide. Like EfficientDet and DETR, these models likely all require better configuration for improved results, as it is surprising these models were so poor compared to the YOLO methods.

### 5.2 Intra‐ and Cross‐Domain Generalisation Results

Our experiments highlight a fundamental discrepancy between controlled training environments and actual clinical scenes (see Tables 3 and 4). These disparities are characterised by higher image resolution, more distinct edges, straightforward scenes with less background motion, lack of occlusion, smoke, glare and other major visual artefacts, all leading to more straightforward scenes. Consequently, while YOLO‐based models can reach near‐perfect instance detection in the surgical training setting, we observed ${\mathrm{mAP}}_{50:95}$ drops of ≈20%, up to ≈50% on in vivo data.

**TABLE 3:** A systematic benchmark of the quantitative laparoscopic tool detection results. The mAP scores are from evaluating the trained models on the held‐out test set of the in‐house dataset. The highlighted most accurate model is in yellow (based on ${\mathrm{mAP}}_{50:95}$, and the fastest is in pink (based on FPS). Overall, we notice the superior performance of the YOLO models, slightly worse performance of the transformer‐based architectures and poorer performance of the EfficientDet and SIMO models. Inference speed correlates positively with model size—larger models have larger (and slower) inference speeds.

Model	Size^a^ ↓	${\mathrm{mAP}}_{50}↑$	${\mathrm{mAP}}_{50:95↑}$	Speed^b^ ↓	FPS ↑	Epochs ↓	TT^c^ ↓	T/E^d^ ↓
Anchor‐based								
YOLOv11‐X [13]	56.9	99.5	95.9	18.8	53	33	4.3	0.13
YOLOv11‐L [13]	25.3	99.5	96.4	9.9	101	49	0.9	0.02
YOLOv11‐M [13]	20.1	99.5	96.1	7.6	132	33	0.5	0.02
YOLOv11‐S [13]	9.4	99.5	94.6	3.3	303	23	**0.2**	**0.01**
YOLOv11‐N [13]	2.6	99.5	94.5	**1.7**	**588**	30	**0.2**	**0.01**
YOLOv10‐X [42]	31.7	98.9	93.5	19.3	52	19	7.8	0.41
YOLOv10‐L [42]	25.8	98.9	89.4	13.0	77	15	3.5	0.23
YOLOv10‐M [42]	16.5	98.2	90.5	8.0	125	**14**	1.6	0.11
YOLOv10‐B [42]	20.5	98.7	92.2	10.3	97	**14**	2.2	0.16
YOLOv10‐S [42]	8.1	99.3	92.7	3.9	256	16	1.8	0.11
YOLOv10‐N [42]	2.7	99.1	93.5	2.2	465	19	1.9	0.10
RetinaNet [43]	36.4	**99.9**	88.3	5.2	192	124	27.9	0.23
EfficientDet [15]	6.6	81.5	58.7	3.5	286	162	3.11	0.02

Anchor‐free								
YOLOv12‐M [20]	20.1	99.5	84.1	6.4	156	30	0.5	0.02
YOLOv12‐S [20]	9.2	99.5	85.0	4.2	238	30	1.5	0.05
YOLOv12‐N [20]	2.6	99.4	84.7	2.1	476	78	1.2	0.02
YOLOv8‐X [44]	**68.2**	99.5	**96.6**	21.7	46	84	18.6	0.22
YOLOv8‐L [44]	43.6	99.5	95.2	12.8	78	36	1.6	0.05
YOLOv8‐M [44]	25.9	99.5	94.5	8.0	125	29	1.7	0.06
YOLOv8‐S [44]	11.1	99.5	96.0	3.4	294	66	1.0	0.02
YOLOv8‐N [44]	3.0	99.5	95.0	1.8	556	41	0.8	0.02
DETR [16]	41.5	90.5	75.3	10.5	95	200	13.9	0.07
DTX [22]	42.0	94.2	85.8	10.6	94	150	10.5	0.07
RT‐DETR‐M [17]	42.0	95.8	87.2	10.8	93	115	8.2	0.07
RT‐DETR‐S [17]	31.0	94.5	85.8	6.8	147	110	5.4	0.05
RT‐DETR‐N [17]	20.0	92.8	83.1	4.2	238	105	3.5	0.03
RF‐DETR‐M [19]	33.7	96.2	88.1	4.5	221	48	2.9	0.06
RF‐DETR‐S [19]	32.1	94.8	84.6	3.5	284	53	3.1	0.06
RF‐DETR‐N [19]	30.5	93.5	82.3	2.3	431	50	2.8	0.06
DINOv3 [21]	121.0	93.5	80.1	34.5	29	80	22.2	0.28
SIMO‐VGG [31]	14.7	82.3	59.2	5.7	175	26	0.9	0.03
SIMO‐ResNet [31]	23.5	83.1	61.4	7.5	133	16	0.8	0.05

^a^ Parameters in millions.

^b^ Inference speed in milliseconds (ms).

^c^ Training time (in h).

^d^ Time per epoch (in h).

**TABLE 4:** Generalisation results. The models we trained on the in‐house dataset were unable to generalise at all on the in vivo datasets. Thus, we trained the same models again on the ART‐Net datasets with its training images, and report their performance on its held‐out test set (in the first column of each mAP metric). In the second and third columns, we report the out‐of‐distribution results from those trained models, without additional fine‐tuning on the EndoVis 2015 dataset (from the trained checkpoint on the ART‐Net dataset, reported in the second column) and the WMU Box‐Trainer dataset (from the trained checkpoint on the EndoVis 2015 dataset, reported in the third column). We include the performance difference in brackets. We do not include some of the worse‐performing models (based on the ${\mathrm{mAP}}_{50:95}$ score), nor all YOLO variants (selecting only the ones we compiled in Table 5).

	${\mathrm{mAP}}_{50}↑$			${\mathrm{mAP}}_{50:95}↑$		
Model	ART‐Net^a^	EndoVis^b^	WMU^c^	ART‐Net^a^	EndoVis^b^	WMU^c^
YOLOv11‐X	88.8	93.3 (+4.5)	74.9 (−24.6)	61.2	64.3 (+3.1)	40.7 (−55.2)
YOLOv11‐M	92.5	95.2 (+2.7)	53.9 (−45.6)	73.9	71.3 (−2.6)	28.3 (−67.8)
YOLOv11‐N	94.9	93.8 (−1.1)	55.6 (−43.9)	80.6	71.2 (−9.4)	29.7 (−64.8)
YOLOv10‐X	83.3	77.2 (−6.1)	**76.5** (−22.4)	61.2	56.9 (−4.3)	44.6 (−48.9)
YOLOv10‐M	77.4	77.8 (+0.4)	61.5 (−36.7)	51.9	51.9 (±0.0)	31.9 (−58.6)
YOLOv10‐N	86.9	92.5 (+5.6)	68.0 (−31.1)	66.8	64.5 (−2.3)	41.1 (−52.4)
RetinaNet	90.1	89.8 (−0.3)	76.4 (−23.5)	59.9	59.6 (−0.3)	**68.0** (−20.3)
YOLOv12‐M	87.0	87.7 (+0.7)	47.5 (−52.0)	74.1	74.7 (+0.6)	23.2 (−60.9)
YOLOv12‐S	91.5	93.0 (+1.5)	54.9 (−44.6)	78.6	79.9 (+1.3)	30.1 (−54.9)
YOLOv12‐N	91.6	93.8 (+2.2)	53.6 (−45.8)	78.5	80.4 (+1.9)	29.6 (−55.1)
YOLOv8‐X	89.2	93.5 (+4.3)	53.2 (−46.3)	70.3	66.2 (−4.1)	31.6 (−65.0)
YOLOv8‐M	89.9	88.9 (−1.0)	69.2 (−30.3)	66.4	65.6 (−0.8)	43.3 (−51.2)
YOLOv8‐N	93.0	95.2 (+2.2)	74.1 (−25.4)	76.7	74.6 (−2.1)	47.7 (−47.3)
DETR	90.5	88.2 (−2.3)	61.1 (−29.4)	75.3	72.8 (−2.5)	38.2 (−37.1)
DTX	94.2	**95.8** (+1.6)	64.2 (−30.0)	85.8	**87.2** (+1.4)	42.1 (−43.7)
RT‐DETR‐M	95.8	94.5 (−1.3)	67.2 (−28.6)	87.2	85.1 (−2.1)	45.5 (−41.7)
RT‐DETR‐S	94.5	92.8 (−1.7)	65.8 (−28.7)	85.8	83.5 (−2.3)	43.2 (−42.6)
RT‐DETR‐N	92.8	90.5 (−2.3)	63.1 (−29.7)	83.1	80.8 (−2.3)	40.8 (−42.3)
RF‐DETR‐M	**96.2**	94.8 (−1.4)	68.5 (−27.7)	**88.1**	86.2 (−1.9)	46.8 (−41.3)
RF‐DETR‐S	94.8	92.5 (−2.3)	66.1 (−28.7)	84.6	82.1 (−2.5)	42.8 (−41.8)
RF‐DETR‐N	93.5	91.2 (−2.3)	64.8 (−28.7)	82.3	79.8 (−2.5)	40.5 (−41.8)
DINOv3	87.7	85.2 (−2.5)	58.1 (−29.6)	68.2	65.5 (−2.7)	35.8 (−32.4)

^a^ Models trained on ART‐Net training images and tested on ART‐Net validation images.

^b^ Models trained on ART‐Net training images and tested on EndoVis 2015 validation images.

^c^ Models trained on in‐house training images and tested on WMU Box‐Trainer validation images.

The methods perform remarkably similarly (better in some cases) on the external validation EndoVis 2015 dataset compared to the ART‐Net dataset. However, there are clear and consistent extreme drops on the WMU dataset, illustrating problems with

(1) $${\text{mAP}}_{50}=\frac{1}{n}\sum_{i=1}^{n}{\text{AP}}_{i}\left(\text{IoU}>0.5\right)$$

(2) $${\text{mAP}}_{50:95}=\frac{1}{41n}\sum_{t=0.5}^{0.95}\sum_{i=1}^{n}{\text{AP}}_{i}\left(\text{IoU}>t\right)$$

more considerable variation in surgical training environments due to specific features which may or may not be present (see Section 3.3). This suggests the over‐fitting problem was due to the data distribution shift rather than the models themselves or training configuration. Thus, models should be trained using various setups with different tools and backgrounds to improve generalisation. We noticed much better performance when fine‐tuning using some sample frames of the WMU dataset (pre‐validation), and are confident in the generalisability of these models, contingent on the distribution of the training data. Nevertheless, due to the lack of generalisation after applying various techniques to reduce over‐fitting, we see models did so anyway and were unable to handle the data distribution shift.

### 5.3 Simulated Resource‐Constrained Results

Table 5 contains the inference speed and accuracy results using the simulated low‐cost hardware^5^. Interestingly, we do not witness a drop in performance through compilation at FP32 and FP16. Thus, we hypothesise three potential explanations: (i) the models may have partially over‐fit on the initial training data. In such cases, the decision boundaries are overly sharp, and quantisation to FP16 does not introduce sufficient perturbation to degrade predictions. Ideally, models should learn just enough structure to generalise; here, excess memorisation may have led to a form of quantisation‐invariance, preserving accuracy despite reduced precision. (ii) The calibration set of 100 randomly‐selected images, while balanced across the dataset, may have been too limited in size or complexity to expose edge cases. If calibration images are overly simple (e.g., clear tool visibility and minimal occlusion), then TensorRT's quantisation ranges are optimised for non‐challenging scenes, which artificially preserves performance. A larger, more diverse calibration subset with complex scenarios (occlusion, blur, atypical lighting) would provide a more robust estimate of quantisation sensitivity. (iii) Even the smallest YOLO Nano variants are already highly expressive relative to the simplicity of the laparoscopic training task, which has low scene variability compared to in vivo surgery. This architectural headroom means that even after precision reduction, the representational capacity remains sufficient. This raises the possibility that even smaller architectures or bespoke, lightweight models could achieve comparable performance at even higher efficiency.

**TABLE 5:** Compilation results of the YOLO models on the NVIDIA Jetson Orin Nano Super Developer Kit (8GB). All values highlighted bold in the speed/FPS sections refer to ‘real‐time’ (>30 FPS), with those bolded and underlined, >60 FPS. The FP64 (64‐bit floating‐point precision) model refers to the original baseline model, without any TensorRT model optimisation techniques, run using maximum power settings with all CPU and GPU cores on the NVIDIA Jetson, for evaluation on the in‐house dataset (thus we see discrepencies in the inference speed on this less powerful hardware). Accuracy results were also obtained using a validation subset, with the highest values in bold. However, since model compilation requires extensive time to calibrate using the validation images (to preserve as much accuracy as possible), only a smaller subset of 100 images was used. Regarding the final size of model weights, the FP16 (16‐bit floating‐point precision) and FP64 (64‐bit floating‐point precision) model were similar, with the FP32 (32‐bit floating‐point precision) generally being twice as large. See Table 2 for more information on the parameter sizes of the different YOLO variants.

	Speed (ms) ↓			FPS ↑			${\mathrm{mAP}}_{50}↑$			${\mathrm{mAP}}_{50:95}↑$		
Model	FP64	FP32	FP16	FP64	FP32	FP16	FP64	FP32	FP16	FP64	FP32	FP16
YOLOv11‐X	456.9	42.9	**20.8**	2.2	23.3	**48.1**	**99.5**	**99.5**	**99.5**	95.9	95.7	95.7
YOLOv10‐X	592.9	41.3	**20.0**	1.7	24.2	**50.0**	98.9	98.0	97.9	93.5	93.5	93.5
YOLOv8‐X	56.9	50.8	**23.5**	17.6	19.7	**42.6**	**99.5**	**99.5**	**99.5**	**96.6**	**96.6**	**96.4**
YOLOv12‐M	181.0	110.3	60.3	5.5	9.1	16.6	**99.5**	**99.5**	**99.5**	84.1	84.1	84.0
YOLOv11‐M	207.2	**18.3**	**9.0**	4.8	**54.6**	**111.1**	**99.5**	**99.5**	**99.5**	96.1	96.1	96.0
YOLOv10‐M	345.8	**17.6**	**9.0**	2.9	**56.8**	**111.1**	98.2	98.2	98.2	89.5	89.5	89.5
YOLOv8‐M	36.1	**18.3**	**9.3**	27.7	**54.6**	**107.5**	**99.5**	**99.5**	**99.5**	89.7	89.7	89.5
YOLOv12‐S	73.3	46.5	**25.1**	13.6	21.5	**39.8**	**99.5**	**99.5**	**99.5**	85.0	85.0	85.0
YOLOv12‐N	39.1	**17.7**	**11.1**	25.6	**56.5**	**90.1**	99.4	99.4	99.4	84.7	84.7	84.7
YOLOv11‐N	97.0	**5.1**	**3.2**	10.3	**196.1**	**312.5**	**99.5**	**99.5**	**99.5**	94.5	94.4	94.2
YOLOv10‐N	109.2	**5.4**	**3.2**	9.2	**185.2**	**312.5**	99.1	99.1	99.1	93.5	93.5	93.4
YOLOv8‐N	**26.6**	**4.9**	**3.1**	**37.6**	**204.1**	**322.6**	**99.5**	**99.5**	**99.5**	94.9	94.9	94.8

### 5.4 Qualitative Results

**FIGURE 3:**
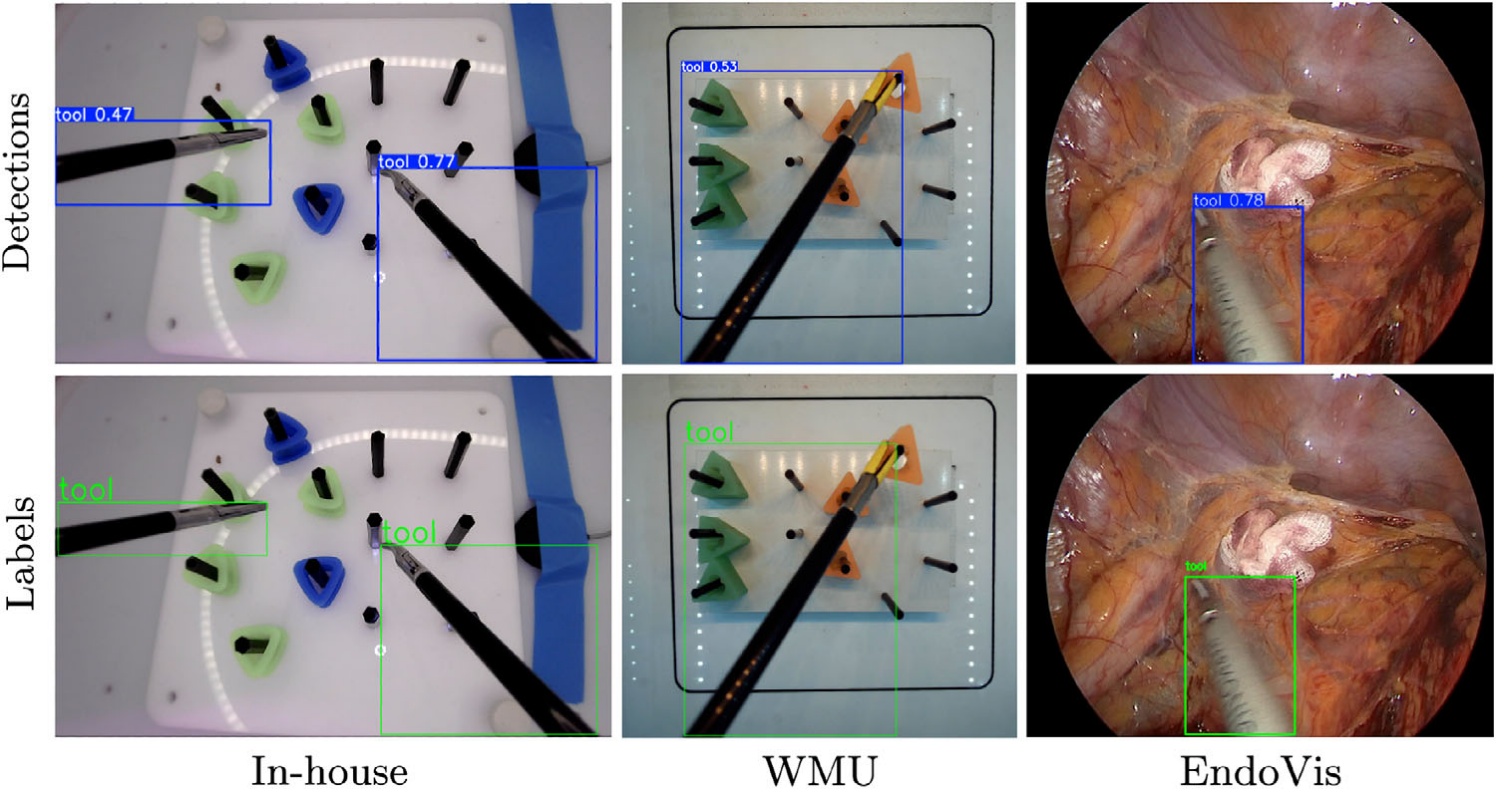
Sample tool detection results using YOLOv8‐X.

Figure 3 shows some sample frames with labelled tools and predictions. Based on Table 3, the given YOLO configurations displayed swift convergence, with a usable model after a handful of epochs. RetinaNet performed best in instances of partial occlusion, overlap, or abnormal aspect ratios, suggesting the anchor‐box optimisation assisted in its performance under challenging examples. In general, we do notice that the YOLOv10 and YOLOv11 models seemed to capture some difficult cases better than YOLOv8, especially when the tooltip was occluded in the scene. The SIMO, EfficientDet and DETR models seemed to approximate the position of the tool in the image but were not very precise with bounding box locations. In transformer‐based models, we see a loss of bounding‐box precision when the tool overlays or contacts the pegs, particularly at the tooltip, due to transient peg‐tool occlusions, specular highlights that flatten local gradients, and similarity in shape/texture, which together make the tooltip boundary ambiguous for attention‐based decoders.

### 5.5 Limitations

Due to occasional tool occlusion, human bias in labelling makes some results challenging to interpret quantitatively. One model may have a slightly worse accuracy score, but it is better when evaluated qualitatively. Not all annotations were performed by the same person, introducing a lack of inter‐annotator agreement. Furthermore, a more detailed hyperparameter search, training‐epoch sensitivity analysis or architectural ablations could have been performed to investigate the non‐YOLO models further. Similarly, a deeper ablation or theoretical insight explaining how specific architectural elements (e.g. heads, attention blocks, or anchor generation) influence the discrepancy of results between anchor‐based and anchor‐free models in the laparoscopic context. In addition, we did not design bespoke architectures tailored specifically for laparoscopic tool detection; instead, we adapted existing general‐purpose object detection frameworks, which may limit optimal performance in this domain (with the exception of DTX as a recent SOTA method in the laparoscopic tool detection task). A balance between architectural innovations to deal with discrepancies in in vivo data should be tested to maintain real‐time performance on constrained hardware.

Moreover, whilst only compilation results of FP32 and FP16 were reported, compilation with INT8 was tested. Whilst it improved inference speeds up to 50% compared to FP16, it resulted in an unfavourable ≈1% reduction in ${\mathrm{mAP}}_{50}$ and ≈20% reduction in ${\mathrm{mAP}}_{50:95}$; hence, these results were discarded. We only compiled the YOLO models due to the simplified compilation process provided by the Ultralytics library [44]. The compilation experiments were further restricted by the use of a single embedded hardware platform, and calibration was conducted on a relatively small validation subset, which may not fully capture accuracy degradation under quantisation. Future work should examine cross‐device reproducibility, energy‐per‐frame measurements, and larger calibration sets to improve the robustness of deployment conclusions.

### 5.6 Conclusion

Despite increasing research on laparoscopic tool detection, few studies articulate how these methods translate to RCEs or tangibly improve surgical training. This paper investigates multiple SOTA object detection architectures on a newly curated in‐house laparoscopic box‐trainer dataset, emphasising real‐time performance on low‐cost embedded devices. We successfully show the potential of standard SOTA models, especially the YOLO series, for exceedingly fast, generalisable and accurate laparoscopic tool detection. We have demonstrated that these methods can be used as backbones for more complex deep learning architectures of surgical skill assessment models for deployment in RCEs to increase and quicken the throughput of surgical trainees. In particular, our findings suggest that the YOLO family provides the most practical balance of accuracy, ease of training, and deployment simplicity, supported by well‐maintained frameworks such as Ultralytics. Compilation with TensorRT was straightforward for YOLO models, highlighting their suitability for integration into embedded workflows. Based on our experiments, the Nano variants (e.g., YOLOv11‐N) consistently achieved performance comparable to larger counterparts but with markedly higher inference speeds. We therefore recommend prioritising nano models in resource‐constrained or embedded contexts, as they maximise throughput without sacrificing accuracy. For training, careful but not exhaustive hyperparameter optimisation was sufficient, indicating that lightweight models can be robustly adapted to laparoscopic domains with limited compute. For deployment, FP16 compilation should be preferred for balancing accuracy and speed, while INT8 may be considered only when extreme efficiency gains are required, given its associated drop in mAP. Overall, the evidence supports adopting lightweight YOLO Nano models as the default configuration for embedded laparoscopic applications, reserving larger models only when domain‐specific accuracy requirements demonstrably justify the latency overhead.

## Author Contributions

All authors contributed to the study conception and design. Software writing was performed by O.C. Data collection was performed by O.C. and D.J. Data analysis was performed by O.C., S.A. and D.J. C.S.B., S.A. and D.J. supervised the research. The manuscript was written by O.C. and all authors commented on previous versions of the manuscript. All authors read and approved the final manuscript.

## Conflicts of Interest

The authors declare no conflicts of interest.

## Ethics Statement

Approval for the dataset collection was granted by the University of Leeds Faculty of Engineering and Physical Sciences Research Ethics Committee (ref: MEEC 22‐023). It contains no personally identifiable information nor human body parts. All other datasets used in this study are entirely non‐identifiable, open‐source, or simulated data and meet the requirements set by the standards at the University of Leeds, so there are no ethical concerns.

## Data Availability

The in‐house dataset will be made publicly available with further data as data collection is still ongoing. All other datasets are publicly available open‐source datasets.

## Endnotes

1 All the frames of the in‐house dataset were from this specific boot camp, as they were annotated prior to further data collection. Our full in‐house dataset comprises 114 videos spanning three boot camps, yet to be annotated [38].

2 We intend to expand this to suturing, circle‐cutting and needle‐passing—tasks from the European Basic Laparoscopic Urological Skills (E‐BLUS) Exam in a larger publicly released dataset in the near future.

3 The original code repository uses the term ‘single input multiple output deep convolution neural network (SIMO‐DCNN)’, trained end–to–end to obtain concurrent outputs.

4 We did also experiment with FP8 but observed a much larger degradation of performance. Due to extensive compilation times on the Jetson per model, we did not complete these for all models and hence not reported.

5 All power settings (15 W, 25 W and maximum power) were tested using both only with the CPU and also with the GPU. Overall, it could be observed that using more power for the device did not significantly impact the inference time, meaning parallel inference could be considered in future work.
